# Value Co-Creation in Participatory Sports Event Tourism: A Mixed Methods Study

**DOI:** 10.3390/bs15030368

**Published:** 2025-03-14

**Authors:** Xiaowei Jiang, Brandon Mastromartino, Xin Li, James J. Zhang

**Affiliations:** 1 College of Physical Education, Chengdu University, Chengdu 610106, China; xinfat@sina.com; 2 L. Robert Payne School of Hospitality and Tourism Management, San Diego State University, San Diego, CA 92182, USA; bmastromartino@sdsu.edu; 3 Department of Kinesiology, University of Georgia, Athens, GA 30602, USA; jamesz48@uga.edu

**Keywords:** sport tourism, event operations, value initiatives, tourism behavior

## Abstract

This study adopts a mixed methods approach to investigate the realization process of value co-creation in participatory sports event tourism by examining the pathways linking predisposing factors, process factors, and outcome factors in event tourists’ value co-creation behaviors. The findings illuminate the unique dimensions of value co-creation in participatory sports event tourism. Governments and event organizers play a pivotal role by proposing value initiatives and fostering co-creation environments through proactive measures, thereby shaping the conditions conducive to co-creation behaviors among event tourists. Embedded in the value co-creation process, tourists’ behaviors are driven by the interplay of their intrinsic motivations and external environmental factors. Their co-creation activities follow patterns of interaction and resource integration, generating diverse experiential values. This study provides innovative strategies for value enhancement to event organizers and offers theoretical insights for governmental governance in sports tourism development.

## 1. Introduction

The construction of a “sports + tourism” industrial chain has become a prominent trend in China, signifying the transformation of the tourism industry from basic sightseeing to advanced operation models and from single-dimensional to multi-dimensional frameworks (Xia et al., 2023). This shift represents a broader movement in tourism that is increasingly driven by experiential value, where consumers seek not only destinations but rich, immersive experiences that resonate with their emotions and preferences (Pine & Gilmore, 2011). Simultaneously, it marks a strategic shift for the sports industry, transitioning from product-oriented manufacturing to service-driven operations. Within this framework, sports act as the core content, while tourism provides the contextual platform (Jiang et al., 2021). The synergy between these sectors generates complementary advantages, fostering integrated innovation that drives growth through experiential offerings, which have become the primary source of added value in today’s tourism market. In recent years, bolstered by favorable policies, the integration of sports and tourism in China has achieved remarkable progress (Feng, 2024). It has emerged as the fastest growing and most dynamic segment within the tourism marketplace (Jiang & Chen, 2023). By 2023, the market size of China’s domestic and international sports tourism sector reached approximately USD 59.72 billion. As the integration of sports and tourism deepens, barriers to entry for social organizations have been lowered, event approval processes have been decentralized, and public enthusiasm for fitness has surged. The COVID-19 pandemic has further heightened public awareness of exercise, health, and quality of life, redefining the value perception of sports tourism and emphasizing immersive experiences (Jiang & Chen, 2023). Within this context, the structure of sports tourism has undergone a reversal where participatory sports event tourism has become the dominant form, while spectator tourism has assumed a secondary role. In sports tourism, the question of “what to do” now plays a more significant role in shaping tourists’ decisions than the question of “where to go”. Participation in sporting events has emerged as a popular leisure and entertainment choice, shifting from passive observation to active involvement. Amid these changes, the supply–demand dynamics and value creation mechanisms within China’s sports tourism market are experiencing profound transformations.

Society has seen a shift where traditional value creation centered on goods is no longer sufficient to meet the evolving demands of the market. Modern consumers are no longer content with merely the functional value of products; instead, they seek diverse and enriched consumption experiences. Moreover, the role of consumers in economic activities has fundamentally transformed-from passive recipients of goods or services to active contributors to value creation, heralding the era of value co-creation (Vargo & Lusch, 2004). While value co-creation has garnered extensive attention in fields such as marketing, service experiences, and customer management, its underlying mechanisms within participatory sports event tourism remain relatively underexplored. This study builds upon existing literature and empirical evidence, employing a mixed methods approach. In this study, we explore the conceptual mechanisms of value co-creation by multiple stakeholders within the context of participatory sports event tourism, shifting from a unidirectional to a multidirectional integration of participants and from a focus on outcome evaluation to a more comprehensive understanding of the value co-creation process.

## 2. Conceptual Background

### 2.1. Participatory Sports Event Tourism

As a blend of sports events, tourism, and active engagement, participatory sports event tourism provides structured competition and recreational opportunities, offering unique embodied experiences. It can be defined as a form of cultural tourism in which individuals actively partake in high-intensity sports events during their leisure time, pursuing competitive, recreational, and health-related benefits. Tourists engage in heightened embodied experiences through intense physical activity, kinesthetic sensations, and proprioception, distinguishing them from the primarily sensory engagement of spectator tourism (Lukanović & Janković, 2024). From a destination perspective, participatory sports event tourism plays a critical role in urban branding, economic growth, and social cohesion (Koutrou & Kohe, 2024; Zhao et al., 2016). From a participant standpoint, Kruger and Saayman (2013) identified five core motivations: social interaction, self-achievement, competition and adventure, reality escape, and commitment. Rejón-Guardia et al. (2020) further categorized mountain bike race participants into three distinct groups, including multifunctional seekers, practical reputation seekers, and sensory seekers, based on motivational dimensions such as physiological sensations, hedonism, and prior experience. Considering China’s sociocultural context, Xing (2016) outlined five key participation drivers: health maintenance, psychological coping, external acceptance, internal fulfillment, and social interaction. Moreover, Hemmonsbey and Maloney (2020) highlighted that destination branding significantly impacts tourists’ length of stay, repeat participation, and willingness to recommend events. In general, key factors influencing the development of participatory sports event tourism include the relationship between the event and the destination, event management efficiency, service marketing capabilities, and stakeholder relationships (S. Wang & Jiang, 2024). 

Research on participatory sports event tourism has gradually moved beyond the singular perspective of sports studies, evolving into an interdisciplinary and multi-sectoral field that integrates insights from various disciplines and domains, leading to the emergence of new conceptual frameworks (Jiang & Chen, 2023). This framework recognizes that participatory sports event tourism shares fundamental characteristics with general tourism activities while possessing unique attributes that distinguish it from conventional tourism models. It emphasizes the deep integration of sports events and tourism destinations as well as the blending of leisure and competitive experiences. More importantly, it highlights the critical role of value co-creation among participants, shaping their engagement and experiences within the participatory sports event tourism context. However, there remains a gap in the literature as comprehensive multidisciplinary systems and a multi-perspective research paradigm have yet to be fully established.

### 2.2. Stakeholders in Participatory Sports Event Tourism

Stakeholder theory emphasizing the shared responsibility of all parties in managing these goals, rather than focusing solely on the contributions and management of individual shareholders (Gomes et al., 2024). Different stakeholders, such as local governments, host communities, and event participants, possess varying capabilities, resource advantages, and information-processing capacities (Söderman, 2023). Jiang and Chen (2023) highlights government departments, event organizers, and event tourists as central stakeholders in participatory sports event tourism, evaluating them through the dimensions of influence, legitimacy, and urgency. Influenced by service-dominant logic, event tourists have emerged as the most direct stakeholders in participatory sports event tourism, assuming an increasingly significant role (Shi & Yang, 2020). Event organizers, as key facilitators, adopt market-oriented strategies, integrate capital and resources, and employ specialized methods to shape the participatory sports event tourism environment and expand value dimensions effectively (Pranata et al., 2023). Meanwhile, government departments, with their crucial role in resource allocation and strategic event management, are indispensable for ensuring the multidimensional development and sustained impact of participatory sports event tourism. Building on the indications of Jiang and Chen (2023) and Shi and Yang (2020), this study employs stakeholder theory to identify governmental departments, event organizers, and event tourists as core stakeholders in participatory event tourism. It examines how their interactions and collaborations directly contribute to value creation within this sector.

### 2.3. Value Co-Creation

Following the traditional product-driven logic, some people may view sports events as consumable goods, often neglecting the active role of event tourists in value creation. Woratschek et al. (2014a) introduced the Service-Dominant Logic (SDL) to the sports context through the Sports Value Framework (SVF), highlighting that value in sports is co-created through stakeholder interactions rather than being independently produced by organizations. In event tourism, value emerges from tourists’ interactions with other stakeholders and resource integration, with sports organizations merely offering value propositions (Woratschek et al., 2014b). The effectiveness of value co-creation depends on stakeholder roles, resource structures, and value propositions (Grohs et al., 2019). Depending on the nature of a participatory sport event and how well it is planned and managed, different sports events can exhibit distinct value co-creation dynamics. In spectator events, athletes provide engaging performances, fans enhance the atmosphere, and sponsors contribute resources while leveraging the platform for brand exposure (Rosca, 2013; Cook et al., 2023). In contrast, participatory sports event tourism emphasizes the dual role of participants as both creators and recipients of the event experience. By actively engaging tourists thorough both self-motivation and organizational initiatives, tourists can reshape event landscapes, enhance collective identity, and foster positive experiences through feedback and customization (Jiang & Chen, 2023; Rejón-Guardia et al., 2020). These interactions not only sustain behavioral intentions but also promote sports culture, drive sports consumption, and expand participation (Solberg et al., 2024).

Although some studies have emphasized the complex relationships among stakeholders in participatory sports event tourism and highlighted the importance of collaborative participation in value creation (Grohs et al., 2019), most studies have focused on the unidirectional integration of actors rather than multidirectional interactions, often failing to depict a comprehensive value co-creation network (Woratschek et al., 2014a). Research on value co-creation in participatory sports event tourism should fully address the industry’s unique and complex characteristics, with particular attention to the coupling processes among diverse participants (Jiang & Chen, 2023). From a systems perspective, this study aims to address two key research questions: (1) How do participants in participatory sports event tourism engage in value co-creation, and (2) What are the interrelationships among the elements of value co-creation? This study seeks to construct a conceptual model that integrates and analyzes the participatory sports event tourism value co-creation process, offering deeper insights into the intrinsic mechanisms driving value co-creation in this context.

## 3. Research Overview

To address the research questions, we employed a mixed methods approach with a two-phase research design (Creswell & Plano Clark, 2007). The qualitative phase focused on developing a process model of value co-creation in participatory sports event tourism, drawing insights from the perspectives of government, event organizers, and event tourists. Using a programmatic grounded theory approach, we conducted data coding in three stages to comprehensively explore the value co-creation process, addressing the first research question. The quantitative phase validated the theoretical model by designing a measurement tool and examining associations among stakeholder behavioral variables, with an emphasis on event tourists, in an effort to answer the second research question.

## 4. Study 1: Qualitative Study

### 4.1. Participants and Data Collection

Following the principles of sample selection, including typicality, completeness, and accessibility, we selected marathon and other road race tourism events in China as case studies. Data were collected from four sources: event documents (e.g., policies and regulations), semi-structured interviews, event field observations, and event management-related research literature. These data were cross-analyzed and mutually verified to build a comprehensive evidence chain. The data collection process lasted for 8 months, resulting in 34 interview samples, 889 min of interview recordings (some interviews were recorded using notes due to lack of recording authorization), 62,000 words of interview transcripts, 3 event observation notes, 41 policy and regulation texts, and 102 event operation documents.

### 4.2. Analyses

**Figure 1:**
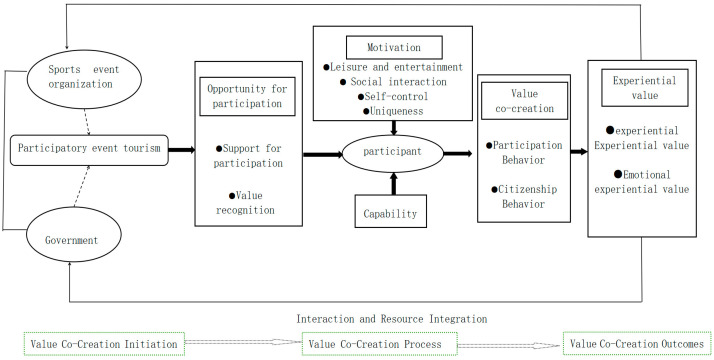
Mechanism model of value co-creation in participatory sports event tourism.

This study adopted programmatic grounded theory, which avoids preconceptions and ensures value neutrality throughout data collection. Through open, axial, and selective coding, we refined and categorized data to develop a cohesive theoretical framework for value co-creation in participatory sports event tourism. Initially, open coding was performed using “literature coding”, “apprenticeship coding”, and “self-created coding”, resulting in 121 initial concepts. These concepts were then integrated based on semantic similarity, forming 21 categories, such as participation support, value recognition, and citizen behavior. We identified relationships between these categories, clustering them to form core categories. Through continuous comparative analysis of these categories, the “process mechanism of multi-stakeholder value co-creation” emerged as the core category. Building upon the Motivation–Opportunity–Ability mode, we established the relational framework for value co-creation in participatory sports event tourism and developed a theoretical model (Figure 1). To test theoretical saturation, we used four sets of interview transcripts and four policy texts. After repeated comparisons and in-depth analysis, the three researchers involved in the coding unanimously concluded that no new concepts or categories emerged, and the internal structure of the storyline remained unchanged. Therefore, we conclude that the model framework has reached theoretical saturation.

### 4.3. Findings

#### 4.3.1. Overall Structural Characteristics of a Value Co-Creation Model

The value co-creation model in participatory sports event tourism can be conceptualized as a complex system in which event organizers, government agencies, and event tourists interact and collaborate, both directly and indirectly, to optimize resource utilization, exchange, and integration. The value co-creation behavior of event tourists results from the combined influence of external environmental drivers and intrinsic motivations. Participation motivation serves as the primary catalyst and internal force driving event tourists’ value co-creation behaviors. Key drivers, such as the need for leisure and entertainment, self-control, social interaction, and uniqueness, transition the value creation process from a passive observation state to an active engagement state. Participation ability refers to a relatively stable internal characteristic of event tourists, reflecting the alignment between their economic, physical, and knowledge-based capabilities and their involvement in value co-creation activities. This ability indicates the goals and outcomes they are likely to achieve during the co-creation process. Participation opportunity is defined by event tourists’ awareness of their current environment, encompassing various social, economic, cultural, and policy factors. This awareness arises from the interaction and collaboration between government entities and event organizers, ultimately providing external support for value co-creation. This support is considered as favorable components derived from the external environment, which stimulate event tourists’ co-creation behaviors.

#### 4.3.2. Process Structure of the Value Co-Creation Model

(1) Antecedent Elements—Initiation of Value Co-Creation and Environmental Creation

In the process of value co-creation throughout participatory sports event tourism, the “initiation” phase signifies the efforts of traditional service providers, such as government bodies and event organizers, to foster development and provision through collaborations, forward thinking, and attention to details. Rooted in service-dominant logic, value is co-created through the exchange of value propositions, rather than direct transfers of resources. Although government entities and organizers pursue different roles and objectives, they share a common goal of delivering value to customers. Involved parties integrate their propositions into a broader service ecosystem, engaging multiple stakeholders to create an environment conducive to co-creation. For the realization of value creation in participatory sports event tourism, coordination and cooperation between government bodies and event organizers are essential. This collaboration encompasses resources, information, and technology—elements necessary to achieve shared goals. By aligning these resources and efforts, the solid foundation for successful value co-creation can be established. When event tourists are attracted to these propositions, they actively respond by contributing resources and engaging in interactions, thereby fostering collaborative dialogues and resource integration. This process mitigates zero-sum dynamics, promotes mutual benefits, and strengthens the potential for co-creating value. As one government official noted in an interview: “Conflicts and challenges inevitably arise, but through collaboration and dialogue, consensus is reached, enabling multi-party value co-creation” (Participant 2).

(2) Process Elements—Participant Embedding and Resource Integration

In the value co-creation process throughout participatory sports event tourism, “event tourist embedding” refers to the integration of event tourists’ motivations and participation abilities within the “field” and “hierarchical structure” of value co-creation, positioning event tourists at the core of this process. When tourists perceive one or more specific needs, such as the “need for leisure and entertainment”, the perception triggers behaviors aimed at fulfilling these needs. As one participant explained: “The pressure from work and life is quite high now, and I don’t spend much time with my family or kids. By participating in this marathon, I can bring my family out to relax and let my kids experience this atmosphere” (Participant 23). However, constraints, such as physical and financial limitations, may influence the fulfillment of these needs. As another participant noted: “I set myself the goal of running three marathons each year, and the amount I can afford to spend is limited, especially since I am still a student relying on family support” (Participant 32). Moreover, interaction and resource integration constitute the behavioral trajectory of value co-creation in participatory sports event tourism. These processes affect the existing value creation network, shaping the operational mechanisms and outcomes of the system. Event tourists, as central participants of value co-creation, usually exhibit different behaviors at various stages of the event, displaying dynamic and diverse actions throughout the event lifecycle (pre-event, during-event, and post-event). Prior to the event, tourists typically seek information from various channels to enhance the rationality of their decisions. These include both formal platforms, such as government websites and event organizers’ social media, as well as informal sources, like promotional materials. During the event, tourists engage with organizers and fellow participants, providing feedback and support. After the event, they continue to co-create value by sharing experiences and recommending the event to others, thus enhancing their social recognition.

(3) Value Generation

Value is realized through its continuous utility, as unimplemented value holds no meaning (Chandra & Rahman, 2024). Event tourists, as active participants, derive use value by transforming, using, and integrating resources, specifically manifesting as experiential value, including functional and emotional experiences. Other stakeholders, like governments and organizers, primarily benefit indirectly from experiential value. Positive interactions and experiences among event tourists foster increased consumption and generate user data and social capital, which provide competitive advantages for event organizations in the data-driven era. Additionally, event tourists’ experiential value creates positive externalities for urban development, enhancing city branding, sports culture, residents’ fitness awareness, and urban resilience. Governments also benefit from spillover effects, gaining comprehensive developmental advantages. As one participant noted: “Chengdu strives to build itself into a city of events, using events to promote national fitness and spread the value of sports” (Participant 3).

Overall, Study 1 explores the value co-creation process in participatory sports event tourism from a holistic, inductive, and qualitative perspective, uncovering the realization and transmission mechanisms of value co-creation at a practical level. The study demonstrates that the foundation and focal point of value creation in participatory sports event tourism lie with event tourists. The core issue in the value co-creation mechanism of participatory sports event tourism is addressing the “human” factor. Value is not merely an entity dependent on the existence of social organizations; rather, it is a subsequent, derivative, and social experiential product centered around “people”, providing a preliminary answer to research question 1. Building on these findings and adopting related theories and previous research findings, a quantitative study was designed to further examine the interrelationships among factors influencing value co-creation in participatory sports event tourism. Specifically, the study investigates whether participation motivation, as core determinants, directly influence event tourists’ value co-creation behavior. Necessarily, this study further explores the moderating roles of participation opportunities and participation capabilities in the relationships. In turn, the study examines whether event tourists’ value co-creation behavior directly impacts multiple value outcomes.

## 5. Study 2: Quantitative Study

### 5.1. Scale Development

The first step involved determining the scope of the constructs, generating a pool of items for the scales, and defining the appropriate response formats. For tourists’ participation motivation (including need for leisure and entertainment, self-control, social interaction, and uniqueness), we drew on the research of Hsieh and Chang (2016), Jiang et al. (2021), and Nambisan and Baron (2007). For participation capability, which is the ability to respond to the invitation of value co-creation (including physical fitness, economic ability, knowledge and skills), we developed theoretical categories based on the grounded theory, referencing the scales of Li (2021) and Liu and Shi (2021). Since participation capability may vary in both meaning and scope and across research contexts, we applied grounded theory coding to analyze the qualitative data and derive a context-specific definition. This qualitative study allowed us to define participation capability in alignment with the particular characteristics of our research setting, ensuring a more precise and contextually relevant conceptualization. For value co-creation behavior, we primarily referred to the study by Yi and Gong (2013), measuring tourists’ value co-creation behavior across two dimensions: participation behavior and civic behavior. In the section on experiential value, based on findings from the qualitative research, we divided it into emotional experiential value and functional experiential value. The measurement items were adapted from the work of Gallarza et al. (2015), Meng (2019), and Xu and Li (2017). For tourists’ participation opportunities, it is the external support of social networks for the value co-creation of tourists (including support for participation and value recognition). Because no established scale was available, all items were developed based on the definition and qualitative research findings. This construct was measured from two aspects: participation support and value recognition. A five-point Likert scale was utilized, with options ranging from 1 “strongly disagree/not important” to 5 “strongly agree/very important”.

To test the content validity, an expert panel comprising three university professors, one event organizer, and one government official reviewed the questionnaire. Their feedback focused on verifying translation accuracy, ensuring item clarity and understandability, and aligning variable measurements with the research content. After revisions based on expert feedback, we finalized the preliminary survey questionnaire. Although this study referenced numerous empirically validated scales, some items were contextually adjusted, and new measurement items were developed based on grounded research content. Following DeVellis’ (2012) suggestion, a small-scale pre-survey was conducted to further enhance the reliability and validity of the questionnaire. This included revising items based on total correlation coefficients and Cronbach’s alpha coefficients, conducting reliability testing, and performing exploratory factor analysis to assess the questionnaire’s validity. The final step in scale development was to optimize the length of each scale by determining the set of items to be included (DeVellis, 2012). The goal was to create concise scales that facilitate responses from the target population while ensuring validity and reliability.

### 5.2. Survey Instrument

Based on the validity and reliability analysis of pre-survey data, the study refined the initial scale, resulting in a final survey scale of 40 items. This included 9 items for participation opportunities, 3 for participation abilities, 12 for participation motivations, 8 for value co-creation behaviors, and 8 for experiential value. A preface section was also added to introduce the study and gather respondents’ personal information, completing the questionnaire’s formal design.

### 5.3. Analysis and Results

#### 5.3.1. Participants and Data Collection

The quantitative study’s data collection involved two stages. A small-scale pilot survey was conducted first to refine the questionnaire based on reliability and validity analysis. The formal survey, carried out between June and October 2023, used online and offline methods, yielding 933 responses, of which 816 were valid after three screening rounds. Following Hair et al.’s (2006) recommendation that SEM requires a sample size of at least 200 or a 1:10 ratio of observed variables to sample size, this study’s 40 observed variables necessitated a minimum of 400 responses. Schwarzer et al. (1997) noted that larger SEM samples may reduce model fit, potentially leading to hypothesis rejection. Thus, the 816 valid responses provide a sufficient balance between parameter estimation accuracy and model fit. Descriptive statistical information for the sample is provided in Table 1.

#### 5.3.2. Measurement Properties

The data were processed using SPSS 20.0, and the results indicated that the corrected item-total correlation (CITC) values for the measurement items ranged from 0.60 to 0.960, all exceeding the minimum threshold of 0.40. Therefore, no items were identified for removal. Additionally, the Cronbach’s alpha values for all variables ranged from 0.848 to 0.976, all surpassing the minimum threshold of 0.50, suggesting that the survey instrument exhibits strong internal consistency. According to Anderson and Gerbing (1988), conducting unidimensionality tests on the variables using survey data is essential for assessing the construct validity of each measurement. In this study, exploratory factor analysis (EFA) was first performed on the data, yielding KMO values greater than 0.7 for all variables, and the cumulative variance explained exceeded 70%. These results indicate that the included items effectively represent the latent constructs (C. Wang et al., 2001). Subsequently, confirmatory factor analysis (CFA) was conducted using AMOS 24.0, and the standardized factor loadings, average variance extracted (AVE), composite reliability (CR), and other fit indices all met the established thresholds. The goodness-of-fit indices also surpassed the critical values (2/df = 2.683, RMR = 0.029, RMSEA = 0.045, GFI = 0.928, TLI = 0.967, IFI = 0.972, NFI = 0.956, CFI = 0.972), confirming that the overall model fit is satisfactory.

#### 5.3.3. Construction of Structural Equation Model (SEM) and Direct Effect Analysis

**Figure 2:**
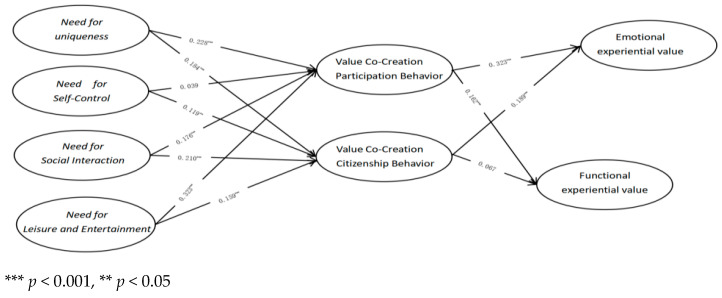
Calculation result of the path coefficient of the structural equation.

The reliability and validity analysis discussed above demonstrates that the measurement of each variable is both reliable and valid, making it suitable for subsequent structural equation modeling (SEM) analysis. The hypothesized model in this study incorporates several latent variables. To facilitate empirical investigation and clarify the logical relationships involved in value co-creation in participatory sports event tourism, we present the core theoretical model (M1) in Figure 2. This model primarily addresses key questions such as: “How do event tourists engage in value co-creation?”, “How do the various elements influence the value co-creation behaviors of event tourists?”, and “What benefits arise from value co-creation?”. The fit indices for model M1 were calculated, with a chi-square to degrees of freedom ratio (2/df) of 2.136, which is below the threshold of 5, indicating a good fit between the model and the sample data. However, since both chi-square values and the 2/df ratio can be influenced by sample size, it is necessary to consider additional fit indices for a comprehensive evaluation (Wheaton, 1987). In this case, RMSEA = 0.037 < 0.05; GFI = 0.922 > 0.9; TLI = 0.962 > 0.9; IFI = 0.966 > 0.9; NFI = 0.938 > 0.9; CFI = 0.966 > 0.9. All of these indices satisfy the criteria for model fit, confirming that the overall model fit is adequate and that the model is suitable for further analysis.

Subsequently, the path coefficients were calculated using AMOS 24.0, as shown in Figure 2. The results reveal that all standardized path coefficients are positive, suggesting that the relationships between the variables are positively influenced. This finding aligns with the results of the qualitative research conducted in Study 1. Furthermore, with respect to significance testing, most path relationships were found to be significant. However, the path coefficient for the relationship between the need for self-control and participation behavior was 0.039, with a *p*-value of 0.253, indicating that this path did not achieve statistical significance. Similarly, the path coefficient for the relationship between civic behavior and functional experience value was 0.067, with a *p*-value of 0.098, which also failed to reach statistical significance.

#### 5.3.4. Moderating Effect Testing

We employed AMOS software to conduct a multi-group analysis of structural equation modeling (SEM), specifically to examine the moderating effects of participation opportunities and participation abilities on the relationship between participation motivation and value co-creation behavior. The process was carried out in the following steps: First, participation opportunities (including participation support and value identification) and participation abilities were transformed into categorical variables, and participants were grouped accordingly. Second, the path coefficients for each group were computed based on the research model, and the significance of the differences in these coefficients was assessed.

(1) The Moderating Role of Participation Opportunities

K-means clustering was applied to the observed variables of participation support and value recognition, with the number of clusters set to two and initial cluster centers not fixed, allowing the algorithm to iteratively refine group assignments based on data patterns. This method ensured an objective classification of participants, reducing potential biases from arbitrary cut-offs. After several iterations, the final results, shown in Table 2, indicated that Group 1 exhibited significantly higher sample means for all observed variables when compared with Group 2. Significance tests further confirmed substantial differences between the groups, and the relatively balanced distribution suggested effective clustering. Consequently, Group 1 was labeled as the high-score group, and Group 2 as the low-score group.

**Figure 3:**
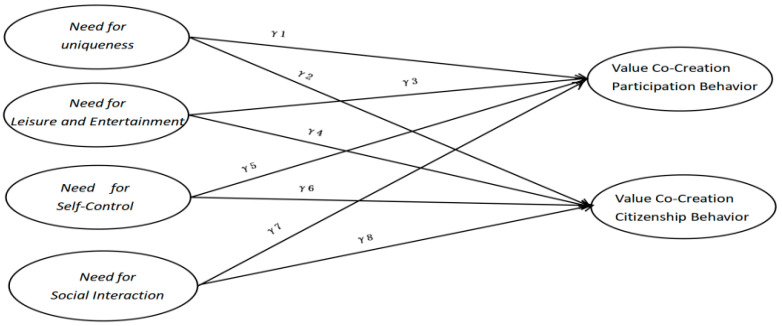
Simplified model of the influence of drivers on value co-creation (M2).

Next, a SEM multi-group analysis was conducted on event tourists’ participation opportunities, using participation support and value recognition as moderating variables. To reduce interference from other variables, the model M1 was simplified by excluding the experiential value variable, resulting in a revised model M2 (Figure 3) focused on the driving factors of value co-creation. Structural equation goodness-of-fit tests were conducted across different categories, estimating path coefficients. The results show that the chi-square/df ratio for the multi-group model was below 5, with RMSEA values below the recommended 0.06. Fit indices, including NFI, CFI, IFI, and RFI, all exceeded 0.90, suggesting good model fit and confirming the theoretical models’ validity. The estimated results are shown in Table 3.

In a multi-group structural equation model analysis, the moderation effect is established only if the following two conditions are met: (1) the path coefficients from the independent variable to the dependent variable exhibit significant differences across different groups; and (2) the magnitude of these path coefficients aligns with the research hypotheses (Redondo & Fierro, 2005). The results clearly illustrate the differences between the different group samples. Following Redondo and Fierro’s (2005) suggestion, differences in standardized path coefficients should not be the sole criterion for significance. The path coefficient differences were further tested through a two-step process: First, the unrestricted model was tested without constraints on path coefficients, and chi-square (χ^2^) and degrees of freedom (df) values were recorded. Then, the path coefficients for the high- and low-score groups were constrained to equality, producing a restricted model, and new χ^2^ and df values were recorded. By comparing these values and calculating changes in χ^2^ (∆χ^2^) and degrees of freedom (∆df), a significant difference (*p* < 0.05) would confirm a moderating effect.

Based on the above steps, five path coefficients showed significant differences at the 0.05 level (Table 4). Specifically, for participation support moderation, the path from need for uniqueness to participation behavior (γ1) and the path from need for social interaction to participation behavior (γ7) were significant. In value recognition moderation, the paths from need for uniqueness to participation behavior (γ9), need for leisure and entertainment to participation behavior (γ11), and need for leisure and entertainment to citizenship behavior (γ12) were significant. These results suggest that increased participation support strengthens the influence of participants’ need for uniqueness and leisure on value co-creation behavior. Value recognition also enhances the impact of the need for uniqueness on citizenship behavior and the effect of leisure and entertainment on both participation and citizenship behavior.

(2) The Moderating Effect of Participation Ability

We continued using a similar method to assess the moderating effect of participation ability. Based on K-means clustering analysis, with the number of clusters set to 2, the three observed variables of participation ability were clustered. After several iterations, Group 1 was labeled as the high participation ability group, consisting of 451 samples, accounting for 55.27% of the total sample. Group 2 was labeled as the low participation ability group, consisting of 365 samples, accounting for 44.73% of the total, with the two groups being relatively evenly distributed, indicating a satisfactory clustering effect.

Subsequently, AMOS software was used to analyze the simplified model M2. The fit indices indicated that the chi-square (χ^2^) value for the high participation ability group was higher than that of the low participation ability group (269.931 > 223.672), with degrees of freedom (df) both being 156. The χ^2^/df values were 1.794 and 1.434, respectively, both below the threshold of 3. Additionally, the RMSEA values for both groups were below the recommended standard of 0.06, and the fit indices, such as NFI, CFI, IFI, and RFI, all exceeded 0.90, suggesting good fit for both sample groups and confirming the acceptability of the theoretical model.

We then performed SEM multi-group analysis to test whether there were significant differences in the path coefficients of the relationship between event tourists’ participation motivation and value co-creation behavior between the two participation ability groups. Among the eight path coefficients in the equality-constrained model for participation ability, three path coefficients showed significant differences at the 0.05 level: the path from need for uniqueness to participation behavior, the path from need for uniqueness to citizenship behavior, and the path from need for social interaction to citizenship behavior. The remaining paths did not pass the significance test. The results indicate that the effect of event tourists’ need for uniqueness on value co-creation participation behavior increases with higher participation ability. At the same time, participation ability strengthens the impact of the need for uniqueness and the need for social interaction on citizenship behavior.

## 6. Discussion

### 6.1. Conclusions

To address research question 1, we have combined theoretical analysis with a localized research perspective and employed the grounded theory approach to systematically examine the process and mechanisms of value co-creation in participatory sports event tourism. From the perspective of event tourists, we identified key concepts, such as participation opportunity, participation motivation, participation ability, value co-creation behavior, and experience value. This aligns with the principles of experience economy, where the creation of value is increasingly driven by the immersive and interactive experiences in which tourists engage during participatory events (Pine & Gilmore, 2011). The findings reveal that when well planned and executed, value co-creation in participatory sports event tourism can be a systematic, contextualized, and dynamic process. It can be understood as a service ecosystem co-constructed by the government, event organizers, and event tourists, where various elements interact and integrate resources to generate value to consumers.

Regarding research question 2, qualitative analysis identified antecedents, processes, and outcomes as the core components of the value co-creation process, clarifying their interactions and enhancing research rigor. The findings showed that although the need for self-control had no significant impact on value co-creation, likely due to the dual nature of participatory sports tourism, the need for uniqueness, social interaction, and leisure were found to positively influence value co-creation, which were consistent with the findings of previous studies (i.e., Yoshida et al., 2014). Participation opportunity moderates value co-creation behavior in that participation support strengthens the link between uniqueness/social interaction and participation behavior, while value identification enhances the link between uniqueness/leisure and citizenship behavior. Participation ability also moderates these relationships although the effects vary. As the previous empirical results indicate, its moderating effects on participatory behavior and civic behavior exhibit heterogeneity. Specifically, the influence of event tourists’ need for uniqueness on value co-creation participation behavior strengthens as their participation ability increases. Meanwhile, participation ability enhances the impact of event tourists’ need for leisure and entertainment and need for social interaction on civic behavior, and vice versa. For outcomes, functional and emotional experience values emerged as key results. Citizenship behavior had no significant impact on functional experience value; yet, as it is voluntary rather than central to value co-creation, it contributed to social value spillover in sports tourism. According to Herzberg’s two-factor theory, this study suggests that participation behavior acts as a “hygiene factor” and citizenship behavior as a “motivator”, with both factors collectively contributing to the development of participatory sports event tourism.

### 6.2. Theoretical Implications

First, this study contributes to the expansion of value co-creation theory. Since the concept of value co-creation was introduced by Prahalad and Ramaswamy (2000), the role of customers within the industrial value chain has become increasingly prominent. Recent international studies have started to explore value co-creation in sports tourism, particularly in countries like the United States and the United Kingdom. However, this area of research is still in its early stages, and a comprehensive theoretical framework has yet to be established. Moreover, due to significant differences in economic, social, cultural, and ideological contexts between China and Western countries, directly applying Western research and theoretical models is not feasible. For instance, China operates under a socialist market economy, where the government’s role is more prominent when compared to the market-driven capitalist economies of Western nations. Additionally, China’s sports industry is still in its developmental stages and lacks the deeply entrenched sports culture of widespread participation that is characteristic of many Western countries. Furthermore, sports-related consumption in China remains relatively low when compared to Western countries where spending on sports is a well-established part of daily life. These disparities make it difficult to directly apply Western theories and models without adapting them to the unique Chinese context. Therefore, this study investigates the theoretical mechanisms of value co-creation within the context of participatory sports event tourism in China, enriching the theoretical system of value co-creation and broadening the theory’s scope and applicability.

Second, this study introduces an innovative research paradigm for participatory sports event tourism. Participatory sports event tourism is a complex and multifaceted social phenomenon, making it challenging to analyze from a single perspective. However, thanks to contributions from scholars with diverse academic backgrounds, research has expanded in scope and perspective. Recent studies have shifted focus towards examining event tourists’ behaviors and their value creation processes. Despite this, research on value co-creation in the context of participatory sports event tourism remains limited. This study constructs a value co-creation system using a mixed methods approach, which spans from conceptual clarification and theoretical development to empirical testing. The innovation of research paradigms in the humanities and social sciences fundamentally involves the development of heterogeneous research methods and perspectives across time and space. From the perspective of the social construction of knowledge, any viewpoint, method, principle, law, or mechanism that offers a breakthrough or creative contribution to existing research can be considered innovative. Therefore, this study can be regarded as introducing a new paradigm in the study of participatory sports event tourism.

### 6.3. Practical Implications

First, this study offers new insights for participatory sports event tourism marketing. In the service economy era, the relationship between customers and enterprises has undergone significant transformations. However, many event organizations have yet to recognize the strategic value of event tourists in participatory sports event tourism marketing, and they rarely develop interactive, holistic value co-creation capabilities. This study integrates and analyzes the value co-creation process in participatory sports event tourism by constructing a clear conceptual model. It reveals that the intrinsic logic of this process lies in creating event tourists’ experience value through multiple collaborations. Specifically, the experience value of event tourists stems from their interaction with the physical and spatial elements of the event venue. Starting with physical movement, interactions between individuals, and between people and their environment transform embodied sensations and emotions into value-related beliefs and emotional attachments. These interactions, in turn, influence subsequent emotional triggers and behavioral intentions, resulting in collective value co-creation by all stakeholders. Positive interactions with event organizations, government bodies, and other stakeholders strengthen group cohesion within the event space, positively affecting the participants’ psychology and positioning them as builders and promoters of the event tourism brand. Therefore, event tourists create value meanings through multi-level interactions and collaborations, which is essential for the sustainable development of participatory sports event tourism. This study aids event organizations in adjusting marketing strategies by adopting targeted approaches to guide and support event tourists in co-creating outstanding experience value, thereby enhancing the overall value of participatory sports event tourism and offering new perspectives on marketing within the Chinese context.

Second, the findings provide preliminary insights for government agencies overseeing sports event tourism; however, they should be considered as part of an ongoing policy discussion rather than definitive conclusions. In China, participatory sports event tourism is an emerging industry that requires continuous governmental and organizational considerations for policy development and adjustment. The dual nature of sports—as both an industry and a public service—calls for a nuanced governance approach. While this study suggests that government involvement may positively influence event tourists’ value co-creation behaviors, the causal mechanisms are complex and warrant further investigation. Existing economic and administrative theories highlight the role of government in addressing market failures and optimizing resource allocation (Gan et al., 2011). Even so, their direct application to participatory sports event tourism should be approached with caution. The findings may serve as a reference for highlighted discussions on government resource allocation. Certainly, the broader effectiveness of policy interventions remains subject to further empirical scrutiny.

The study results highlight the central role of event tourists in shaping and creating experiential value. By engaging deeply and utilizing their own abilities, they enhance the exchange and use values of participatory sports event tourism products, deriving enjoyment and a sense of accomplishment. Event tourists also generate unique situational value, which subsequently triggers the realization of social and economic values. This aligns with the customer experience logic proposed by Prahalad and Ramaswamy (2000). As an innovative product of mass tourism, participatory sports event tourism has infused new cultural vitality into both service content and practice forms. Compared to traditional travelers, event tourists possess stronger interactive participation abilities and higher expectations for service quality, design details, and unique experiences. The findings suggest that event tourist roles can be enriched through scenario creation, intensifying both physical and emotional interactions. This approach effectively connects event tourist resources and stimulates their enthusiasm for co-creation. Moreover, closely linking event tourists within the same micro-context, with event tourism activities, unique services, and derivative festival events as common focal points, creates a larger space for interaction and engagement.

### 6.4. Limitations and Future Research

This study has some limitations which will require refinement in future research. First, the research subjects are somewhat limited in scope. While this study covers the most widely participated types of participatory sports event tourism, including marathons, triathlons, spartan races, and cycling events, other forms, such as rock climbing events, and canyoning competitions, were not included. Different types of participatory sports events may involve heterogeneous mechanisms of value co-creation, and the theoretical model of this study primarily seeks to identify common patterns, which may overlook the unique characteristics of certain sub-categories of events. Second, due to the limited body of research on value co-creation in participatory sports event tourism in China, there is no universally applicable or consistent measurement scale. Therefore, based on a review of the literature, this study adapted existing value co-creation scales from both domestic and international contexts, and developed a measurement scale specifically for participatory sports event tourism, incorporating findings from qualitative research. Although the empirical results show that the questionnaire and measurement items demonstrate good reliability and validity, there is still the potential for result errors. Third, this study originally planned to conduct field research at multiple participatory sports events, but due to the recurring pandemic, this was not possible. Most of the in-depth interviews were conducted online, which limited their depth and duration. Although supplementary data were collected through text materials, policy documents, and online sources, some factors influencing value co-creation in participatory sports event tourism may have been missed. More field visits and in-person interviews could have potentially uncovered additional valuable insights.

Overall, this study has developed a comprehensive theoretical model of value co-creation in participatory sports event tourism, which has been empirically validated and analyzed, yielding several innovative research outcomes. These contributions help enhance both theoretical understanding and practical applications in the field. However, this study represents only the initial step in this topic area of research. Future studies should build on these findings and focus on several key areas. First, future research could expand the scope of the study by incorporating a more diverse range of participatory sports event tourism contexts. By applying the proposed model to different event types, such as adventure races, community-based running events, and extreme sports festivals, researchers can assess the model’s generalizability and refine its measurement scales, thereby enhancing the robustness and applicability of the findings. Additionally, cross-cultural comparisons could explore how value co-creation mechanisms vary across countries, considering differences in cultural attitudes toward sports participation, government support, and tourism infrastructure. Second, future studies could segment participants based on their level of involvement and sociodemographic background such as age, gender, family income, and occupation to provide a more nuanced understanding of value co-creation behaviors. For example, the experiential value perceived by professional athletes versus amateur participants may differ significantly, affecting their engagement in co-creation processes. Similarly, analyzing the role of spectators who engage in event-driven tourism, such as marathon supporters or family members of youth sports participants, could extend the model to include indirect co-creators, further enriching the theoretical framework. Third, expanding research dimensions by exploring new and emerging types of participatory sports event tourism could provide valuable insights. Events specifically designed for children and adolescents, such as the Kirin Little Warrior race, present unique dynamics where the participants and payers (e.g., parents) are distinct. These events align with the experience economy framework, where not only the physical participation but also the emotional and educational value of the experience influences decision-making and satisfaction. Examining how parents and young participants co-create value through aspects like emotional investment, social interactions, and brand engagement could offer new perspectives on multi-stakeholder value co-creation in sports tourism. Finally, future studies could explore the application of the value co-creation model in other immersive and experiential tourism formats, such as sports heritage tourism (e.g., visiting Olympic sites or hall of fame museums), e-sports tourism, or wellness-oriented sports retreats. These tourism experiences share common experiential attributes with participatory sports events and could further validate the applicability of the model across broader tourism sectors.

## Figures and Tables

**Table 1:** Demographic information of formal survey samples.

Indicator	Category	Number	Percentage (%)
Gender	Male	426	52.20%
	Female	390	47.80%
Age	16–20	59	7.20%
	21–30	229	28.10%
	31–40	302	37.00%
	41–50	194	23.80%
	51–60	21	2.60%
	Over 60	11	1.30%
Educational background	Junior high school and below	61	7.50%
	Senior high school	170	20.80%
	Junior college	249	30.50%
	Bachelor’s degree	267	32.70%
	Master’s degree or above	69	8.50%
Occupation	Student	107	13.10%
	Teacher	94	11.50%
	Worker	70	8.60%
	Employee	320	39.20%
	Self-employed	109	13.40%
	Government staff	82	10.00%
	Other	34	4.20%
Monthly income (RMB)	0–3000	150	18.40%
	3001–5000	217	26.60%
	5001–8000	302	37.00%
	8001–10,000	103	12.60%
	Over 10,000	44	5.40%

**Table 2:** K-means clustering analysis results for participation opportunities.

Observed Variable	Sample Mean ± Standard Deviation		Significance Test	
	Group 1 (*n* = 429)	Group 2 (*n* = 387)	F	*p*
Participation support 1	4.17 ± 0.76	1.79 ± 0.71	2139.801	0.000 ***
Participation support 2	4.13 ± 0.77	1.81 ± 0.67	2071.356	0.000 ***
Participation support 3	4.15 ± 0.75	1.85 ± 0.71	2008.455	0.000 ***
Participation support 4	4.26 ± 0.72	1.91 ± 0.74	2095.068	0.000 ***
Participation support 5	4.03 ± 0.89	1.78 ± 0.72	1536.032	0.000 ***
**Observed Variable**	**Sample Mean ± Standard Deviation**		**Significance Test**	
	**Group 1 (*n* = 417)**	**Group 2 (*n* = 399)**	**F**	***p***
Value recognition 1	3.91 ± 0.81	1.77 ± 0.74	1530.594	0.000 ***
Value recognition 2	3.91 ± 0.79	1.68 ± 0.71	1792.041	0.000 ***
Value recognition 3	3.86 ± 0.80	1.67 ± 0.72	1692.482	0.000 ***
Value recognition 4	3.93 ± 0.72	1.75 ± 0.69	1946.317	0.000 ***

*** *p* < 0.001.

**Table 3:** Path relationship test results of participation opportunities.

Path	High Participation Support		Low Participation Support	
	Standardization Coefficient	*p*	Standardization Coefficient	*p*
Uniqueness → participation behavior (γ1)	0.296	***	0.1	0.143
Uniqueness → citizenship behavior (γ2)	0.183	**	0.142	**
Leisure & entertainment → participation behavior (γ3)	0.380	***	0.237	***
Leisure & entertainment → citizenship behavior (γ4)	0.208	***	0.071	0.31
Self-control → participation behavior (γ5)	0.003	0.944	0.077	0.131
Self-control → citizenship behavior (γ6)	0.107	**	0.119	**
Social interaction → participation behavior (γ7)	0.270	***	0.096	0.059
Social interaction → citizenship behavior (γ8)	0.146	**	0.263	***
**Path**	**High Value Identification**		**Low Value Identification**	
	**Standardization Coefficient**	***p***	**Standardization Coefficient**	***p***
Uniqueness → participation behavior (γ9)	0.293	***	0.109	0.095
Uniqueness → citizenship behavior (γ10)	0.128	**	0.207	**
Leisure & entertainment → participation behavior (γ11)	0.405	***	0.209	**
Leisure & entertainment → citizenship behavior (γ12)	0.229	***	0.053	0.419
Self-control → participation behavior (γ13)	0.006	0.899	0.064	0.221
Self-control → citizenship behavior (γ14)	0.065	0.206	0.157	**
Social interaction → participation behavior (γ15)	0.115	**	0.258	***
Social interaction → citizenship behavior (γ16)	0.179	**	0.220	***

*** *p* < 0.001, ** *p* < 0.05.

**Table 4:** The significance test of participation opportunities moderation.

Participation Support		χ2	df	△χ2	△df	*p*
Unrestricted Model		574.261	316	—	—	—
Constrained Model	γ1 equality	580.557	317	6.296	1	0.012
	γ2 equality	574.768	317	0.507	1	0.476
	γ3 equality	576.221	317	1.96	1	0.162
	γ4 equality	576.41	317	2.149	1	0.143
	γ5 equality	575.592	317	1.331	1	0.249
	γ6 equality	574.392	317	0.131	1	0.717
	γ7 equality	579.718	317	5.457	1	0.019
	γ8 equality	576.376	317	2.115	1	0.146
**Value identification**		**χ** **2**	**df**	**△χ** **2**	**△** **df**	***p***
**Unrestricted Model**		**590**	**316**	**—**	**—**	**—**
Constrained Model	γ9 equality	596.613	317	6.613	1	0.010
	γ10 equality	590.226	317	0.226	1	0.635
	γ11 equality	595.833	317	5.833	1	0.016
	γ12 equality	594.33	317	4.33	1	0.037
	γ13 equality	590.623	317	0.623	1	0.430
	γ14 equality	591.256	317	1.256	1	0.262
	γ15 equality	593.717	317	3.717	1	0.054
	γ16 equality	590.258	317	0.258	1	0.611

## Data Availability

The data that support the findings of this study are available from the corresponding author upon reasonable request.
